# Experimental study on antler powder/chitosan/β-glycerophosphate/polyvinyl alcohol composite platelet-rich fibrin scaffold for repairing periodontal bone defects in rabbits

**DOI:** 10.3389/fbioe.2026.1801650

**Published:** 2026-06-23

**Authors:** Adilai Abula, Kudelaiti Abudukelimu, Yueying Pan, Zilalai Ainiwaer, Boling Li, Xin Yuan, Hongbin Dong, Maihefuzi Aishan

**Affiliations:** 1 Department of Prosthodontics and Implantology, The First Affiliated Hospital (Affiliated Stomatological Hospital) of Xinjiang Medical University, Urumqi, China; 2 School of Stomatology, Xinjiang Medical University, Urumqi, China

**Keywords:** antler powder, chitosan, hydrogel, periodontal regeneration, platelet-rich fibrin, tissue engineering, β-glycerophosphate

## Abstract

**Objective:**

This study evaluated and compared the efficacy of five scaffold materials—AP (Antler Powder)/CS (Chitosan)/β-GP (β-Glycerophosphate)/PVA (Polyvinyl Alcohol) composite scaffold with Platelet-Rich Fibrin (PRF), the same scaffold without PRF, an AP/PVA scaffold, PRF gel alone, and Bio-Oss® bone powder—in repairing periodontal bone defects and promoting periodontal tissue regeneration.

**Methods:**

Fifty-four New Zealand White rabbits were randomly divided into six groups (n = 9/group). A unilateral periodontal bone defect model was established in each rabbit. The defects were treated as follows: (1) AP/CS/β-GP/PVA-PRF composite scaffold; (2) AP/CS/β-GP/PVA scaffold; (3) AP/PVA scaffold; (4) PRF gel; (5) Bio-Oss® (positive control); and (6) no implantation (blank control). Three rabbits per group were sacrificed at 4, 8, and 12 weeks postoperatively. Outcomes were assessed *via* gross observation, radiographic imaging, and histological analysis. Quantitative measurements included newly formed alveolar bone area, periodontal ligament width, and mineralization degree within the defect area.

**Results:**

Gross observation revealed normal gingival color, contour, and texture, with probing depths ≤1 mm in all implanted groups, including the Bio-Oss® group. In contrast, the blank control group exhibited reduced alveolar bone height and 2–3 mm of gingival recession by 8 weeks. Radiographic analysis at 4, 8, and 12 weeks demonstrated continuous bone regeneration in all implanted groups, which showed significantly superior repair outcomes compared to the blank control (P < 0.05). Histologically, all implanted groups displayed the formation of cementum-like tissue, periodontal ligament-like fibers, and alveolar bone-like tissue on the root surface, with progressive maturation over time. Statistical analysis (one-way ANOVA with *post hoc* tests) indicated that at 12 weeks, the AP/CS/β-GP/PVA-PRF composite scaffold achieved significantly better regenerative outcomes than the blank control, the Bio-Oss® positive control, and the other three experimental groups (P < 0.05).

**Conclusion:**

The AP/CS/β-GP/PVA-PRF composite tissue-engineered scaffold significantly promotes periodontal tissue regeneration and bone mineralization in periodontal bone defects, which shows good clinical application potential for periodontal defect repair.

## 1 Introduction

Periodontal disease is a widespread global oral health problem and the leading cause of tooth loss in adults. Its progression is characterized by gradual destruction of the periodontal supporting tissues: as the pathological process continues, periodontal attachment tissues progressively lose their physiological function, ultimately resulting in tooth mobility and eventual exfoliation (Kinane et al., 2017). The ultimate goal of periodontal therapy is to regenerate periodontal tissues—including alveolar bone, the periodontal ligament, and cementum—and to restore their normal physiological function (Sculean et al., 2015). Accordingly, achieving periodontal tissue reconstruction and functional regeneration has long been a major focus and challenge in clinical research.

Substantial research has been conducted in periodontal bone grafting and guided tissue regeneration, yielding notable progress. Periodontal bone grafting refers to the use of graft materials to promote new bone formation, thereby achieving bone regeneration and new attachment (Abushama and Osman, 2024). Currently, four main approaches are commonly used for tissue repair in clinical practice: autogenous or autografts, allografts, xenografts and alloplasts. Autologous bone grafting is considered the gold standard for bone repair materials (Sheikh et al., 2017) and involves using the patient’s own bone tissue to restore alveolar bone defects. However, autologous block grafting has several limitations: it requires an additional surgical procedure, which increases operative complexity and risk, and the donor site may experience discomfort, pain, or infection (Schmidt, 2021). Compared with autografts, allografts carry potential risks of immune rejection (Sohn and Oh, 2019), and generally have low patient acceptance (Fernández et al., 2015; Bucchi et al., 2019).

In recent years, bone tissue engineering (BTE) has played an increasingly important role in the treatment of periodontal bone defects (Augustine et al., 2024). By integrating materials science, cell biology, and growth-factor technologies, BTE aims to repair bone tissue defects. The core components of BTE include scaffold materials, seed cells, and growth factors (Sukpaita et al., 2021); among these, scaffolds must be integrated with various bioactive substances, such as cells, drugs, proteins, and other bioactive molecules, to enhance the efficiency of bone formation (Dong et al., 2024).

Hydrogels have emerged as a new class of functional polymeric biomaterials in recent years. They are crosslinked, three-dimensional hydrophilic polymer networks and offer advantages over traditional materials, including softness, strong resistance to deformation, high water absorption, responsiveness (“smart” behavior), high drug utilization, safety, and convenience (Guo et al., 2024). Hydrogels consist of hydrophilic polymer chains crosslinked through diverse interactions, such as covalent bonds, hydrogen bonding, and van der Waals forces (Hameed et al., 2024). The presence of hydrophilic groups enables rapid water uptake, high water retention, and swelling without dissolution. This distinctive structure confers flexibility, allowing hydrogels to mimic the tissue microenvironment, provide structural support at defect sites, and promote repair of bone defects through intrinsic healing mechanisms (Wong et al., 2023).

Xu et al. (2019) developed a thermosensitive hydrogel composed of chitosan (CS), β-glycerophosphate sodium (β-GP), and gelatin. This hydrogel is a novel drug carrier with a simple preparation process and good biocompatibility. Owing to their excellent material properties and similarity to the extracellular matrix, CS/β-GP hydrogels have been widely used in tissue engineering (Pankongadisak and Suwantong, 2018; Arpornmaeklong et al., 2023; Badr et al., 2023). Polyvinyl alcohol (PVA) is a water-soluble polymer obtained by hydrolyzing polyvinyl acetate. It has high water absorption, degradability, good biocompatibility, and favorable mechanical properties. Through composite cross-linking, a double-network hydrogel can be constructed, which balances high mechanical properties and further enhances the efficiency of cell adhesion and proliferation on the composite scaffold (Rahman Khan and Rumon, 2025).

Antler powder is produced by ultralow-temperature pulverization of ossified sika deer antlers, which preserves the nutritional constituents of antler tissue. The core active components responsible for its regenerative effect are antler-derived peptides, type I collagen, calcium-phosphorus inorganic minerals, and growth factors such as IGF-I and TGF-β1. Among them, antler-derived peptides and type I collagen drive bone regeneration by promoting the proliferation of osteogenesis-related cells and upregulating the expression of osteogenic markers such as ALP and BMP7, calcium-phosphorus inorganic minerals provide the material basis for mineralization, and IGF-I and TGF-β1 synergistically enhance tissue regeneration efficiency by regulating the regenerative microenvironment. As a natural osseous material, its antler-derived peptides exhibit osteogenic activity (Yang et al., 2012; Yoshida et al., 2015; Li et al., 2016; Wang Z. et al., 2024). Moreover, antlers can fully regenerate, avoiding animal-ethics concerns associated with slaughter (Huo et al., 2014). Through a series of studies, Zhang et al. (2012) demonstrated that calcined antler cancellous bone is comparable to calcined human cancellous bone in morphology, pore size, structure, chemical composition, and crystal structure, and can effectively promote the repair of bone defects, suggesting its potential as a novel xenogeneic bone scaffold material.

Platelet-rich fibrin (PRF) is a second-generation platelet concentrate first reported by the French researcher Choukroun in 2000. It consists of a polymerized fibrin matrix that entraps platelets, leukocytes, growth factors, circulating stem cells, and other components (Choukroun et al., 2006). Notably, PRF exhibits remarkable regenerative potential suitable for bone and periodontal tissue repair: its fibrin network acts as a natural three-dimensional scaffold to support the adhesion, migration and proliferation of osteoblasts and mesenchymal stem cells (MSCs). Platelets trapped in the matrix can continuously release physiological doses of growth factors, including platelet-derived growth factor (PDGF), transforming growth factor-β1 (TGF-β1), vascular endothelial growth factor (VEGF), and insulin-like growth factor (IGF) (Jia et al., 2024). PRF is rich in soluble growth factors, which can induce the proliferation and migration of periodontal ligament cells and osteoblasts, accelerate the formation of cementum and alveolar bone, and inhibit the excessive growth of oral epithelial cells (Sneha et al., 2021). In addition, PRF not only helps prevent infection and attenuate inflammatory responses, but also promotes tissue healing and regeneration, facilitates connective tissue regeneration, and supports new attachment formation (Goswami et al., 2024). Compared with exogenous growth factors, PRF-derived growth factors are autologous, avoiding immune rejection and side effects caused by improper dosages, thus serving as an ideal bioactive component for composite scaffolds.

In the search for an ideal bone substitute material, our group (Abudukelimu, 2024) used the above materials to fabricate hydrogel scaffolds with different ratios of antler powder (AP)/chitosan/β-glycerophosphate sodium (CS/β-GP)/PVA. After characterizing their properties and conducting *in vitro* experiments, we evaluated the composite scaffolds in terms of mechanical performance, cytotoxicity, cell proliferation, and their ability to induce osteogenic differentiation of MC3T3-E1 cells. We successfully synthesized three-dimensional hydrogel scaffolds with a loose, porous architecture that provides an optimal microenvironment for osteoblasts and markedly enhances their activity. These findings confirmed that the composite hydrogel scaffolds effectively promote osteoblast proliferation and differentiation.

Building on these preliminary results and aiming to further improve periodontal tissue regeneration, the present study incorporated PRF, rich in growth factors, into the AP/CS/β-GP/PVA composite hydrogel scaffold. We sought to investigate the feasibility of this growth factor-enriched composite scaffold for clinical application.

## 2 Materials and methods

### 2.1 Experimental animals

Fifty-four healthy, 3-month-old male New Zealand White rabbits (conventional grade), weighing 2.2–2.5 kg, were provided by the Animal Experimental Center of Xinjiang Medical University. The study was approved by the Institutional Animal Ethics Committee of Xinjiang Medical University (approval No.: IACUC-JI-20240228-37).

#### 2.1.1 Inclusion criteria

- Healthy 3-month-old male New Zealand White rabbits;
- Body weight ranging from 2.2 to 2.5 kg;
- Sound mental state, intact oral mucosa, and absence of systemic or oral diseases;
- Normal food intake and good adaptability after 1 week of preoperative adaptive feeding.

#### 2.1.2 Exclusion criteria

- Poor general condition accompanied by clinical symptoms such as infection, fever, or diarrhea;
- Oral ulcers, periodontal lesions, or maxillofacial developmental abnormalities;
- A body weight loss exceeding 10% during the experimental period;
- Intraoperative or postoperative death, or inability to complete the experiment due to surgical complications.

#### 2.1.3 Animal screening procedure

After arrival, all rabbits were housed in a standardized environment (temperature 22 °C–25 °C, humidity 50%–60%, 12 h light/dark cycle) for 1 week of adaptive feeding. General conditions including mental status, diet, body weight, and oral mucosa were observed daily. Rabbits that met all inclusion criteria and none of the exclusion criteria were enrolled in the formal experiment.

### 2.2 Experimental grouping

This experiment was a randomized controlled animal study. A total of 54 three-month-old New Zealand White rabbits were divided into 6 groups with 9 rabbits in each group using the random number table method. There were no statistically significant differences in baseline data (body weight, age) among all groups (*P* > 0.05), indicating good comparability.

The specific grouping was as follows:

Group 1 (experimental group): AP/CS/β-GP/PVA composite PRF scaffold material implantation group;
Group 2 (experimental control group 1): AP/CS/β-GP/PVA scaffold material implantation group;
Group 3 (experimental control group 2): PRF gel implantation group;
Group 4 (experimental control group 3): AP/PVA scaffold material implantation group;
Group 5 (positive control group): Bio-Oss® bone powder implantation group;
Group 6 (blank control group): only periodontal bone defect was prepared without implantation of any material.

### 2.3 Major equipment and materials

#### 2.3.1 Animals, drugs, and biomaterials

Fifty-four healthy New Zealand White rabbits were used in this study. Anesthesia was induced using Sumianxin II injection (1.5 mL/vial; Veterinary Research Institute of Changchun University of Military Supplies, Changchun, China), and postoperative infection was prevented with Benzylpenicillin Sodium for Injection (800,000 U/vial; Sichuan Boteng Animal Pharmaceutical Co., Ltd., Chengdu, China). The experimental scaffold materials consisted of deer antler powder (100-mesh ultrafine powder; Tefeng Pharmaceutical Co., Ltd., China), sodium β-glycerophosphate (β-GP, analytical grade, purity ≥98%; Shanghai Yuanye Bio-Technology Co., Ltd., Shanghai, China), polyvinyl alcohol (PVA, molecular weight 1750 ± 50; Aladdin Reagent Co., Ltd., Shanghai, China), and chitosan (CS, degree of deacetylation ≥95%; Zhejiang Jinke Pharmaceutical Co., Ltd., China). Platelet-Rich Fibrin (PRF) was prepared autologously from rabbit blood. As the positive control, commercially available Bio-Oss® bone grafting material (Geistlich Pharma AG, Wolhusen, Switzerland) was used.

#### 2.3.2 Instrumentation and reagents

Surgical procedures were perfov 2′rmed using a low-speed bone drill (Aesculap Acculan 3Ti; B. Braun Aesculap AG, Tutlingen, Germany) and a standard dental extraction kit (Jinzong brand; Shanghai Jinzong Medical Devices Co., Ltd., Shanghai, China). General surgical instruments (scalpels, needle holders, forceps, tissue scissors, periodontal curettes, and periosteal elevators) and Ethicon sutures were supplied by the Experimental Center of the School of Stomatology, Xinjiang Medical University. Radiographic imaging was conducted using a dental X-ray unit (MSD-015; Shanghai Medical Devices Co., Ltd., Shanghai, China), and gross photographs were captured with a Canon EOS-1DX digital camera (18 megapixels; Canon Inc., Tokyo, Japan).

For sample processing and histological analysis, the following equipment was used: a high-speed refrigerated centrifuge (Eppendorf 5804R; Eppendorf AG, Hamburg, Germany), an autoclave (SANYO MLS-3781L-PC; SANYO Electric Co., Ltd., Kyoto, Japan), an −80 °C ultralow-temperature freezer (NuAire NU-99586JE; NuAire Inc., Plymouth, MN, United States), a thermostatic water bath (HH-S2; Nanjing Scientific Instrument Co., Ltd., Nanjing, China), a thermostatic magnetic stirrer (MS-H-S heating type; Dragon Lab Instruments Co., Ltd., Beijing, China), a freeze dryer (Christ Alpha 1-4 LSC; Martin Christ Gefriertrocknungsanlagen GmbH, Osterode am Harz, Germany), and a microtome (Leica RM 2135; Leica Biosystems GmbH, Wetzlar, Germany). Histological sections were observed under an optical microscope (XSP-8CA; Shanghai Optical Instrument Factory, Shanghai, China) and stained using an HE staining kit (Zhongshan Jinqiao Biotechnology Co., Ltd., Beijing, China). Graded dehydration reagents (ethanol and xylene) were obtained from the Stem Cell Laboratory of the First Affiliated Hospital of Xinjiang Medical University.

### 2.4 Methods

#### 2.4.1 Preparation of the AP/CS/β-GP/PVA scaffold

##### 2.4.1.1 Preparation of the chitosan/β-glycerophosphate hydrogel (CS/β-GP hydrogel)

An appropriate volume of glacial acetic acid (0.572 mL, AR grade) was diluted with deionized water to prepare 0.1 mol/L acetic acid solution, which was stored at 4 °C until use. Chitosan (2.5 g) was added to 50 mL of 0.1 mol/L acetic acid solution and stirred with a magnetic stirrer until completely dissolved to obtain a chitosan solution. The solution was then refrigerated at 4 °C overnight for subsequent use.

**FIGURE 1:**
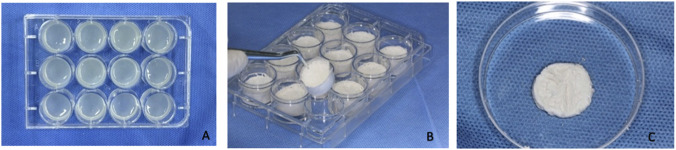
**(A)** chitosan/β-glycerophosphate sodium (CS/β-GP) hydrogel **(B)** antler powder/polyvinyl alcohol (AP/PVA) **(C)** antler powder/chitosan/β-glycerophosphate sodium/polyvinyl alcohol (AP/CS/β-GP/PVA) hydrogel scaffold.

Separately, β-glycerophosphate sodium (β-GP) powder (2.5 g) was dissolved in 10 mL of deionized water with magnetic stirring until fully dissolved, and the resulting β-GP solution was stored at 4 °C. Under magnetic stirring, 10 mL of the β-GP solution was added dropwise to 10 mL of the chitosan solution. After the addition was complete, the mixture was stirred for an additional 30 min (Figure 1A).

##### 2.4.1.2 Preparation of antler powder/polyvinyl alcohol (AP/PVA)

Dissolve 8 g of polyvinyl alcohol (PVA, 1750 ± 50) in 50 mL of deionized water and stir continuously at 90 °C for 4 h to prepare the PVA solution. Then, add 8 g of antler powder (AP) to the PVA solution and stir for an additional 2 h to ensure thorough mixing (Figure 1B).

##### 2.4.1.3 Preparation of AP/CS/β-GP/PVA hydrogel scaffold

The AP/CS/β-GP/PVA hydrogel scaffold was prepared by blending CS/β-GP hydrogel and AP/PVA in a 1:1 volume ratio. The mixture was stirred under ultrasonic treatment until homogeneous, then poured into molds (12-well plates) and placed at −80 °C for overnight pre-freezing. After the pre-freezing, each frozen sample was subjected to freeze-drying for 24 h (Figure 1C).

#### 2.4.2 Preparation of PRF

**FIGURE 2:**
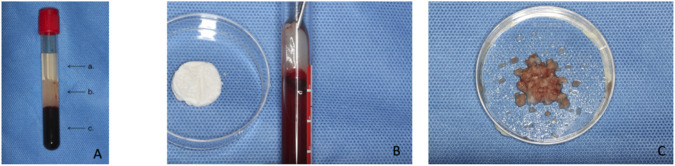
**(A)** Platelet-Rich Fibrin (PRF), **(A)** Serum Layer, **(B)** PRF Gel Layer, **(C)** Blood Cell Layer **(B)** AP/CS/β-GP/PVA Hydrogel Scaffold and PRF Gel **(C)** AP/CS/β-GP/PVA Composite PRF Hydrogel Scaffold.

10 mL of autologous blood was drawn from the ear marginal vein of each rabbit and quickly placed in a centrifuge. The centrifugation was performed at 3,000 rpm for 13 min, without adding any anticoagulant or coagulant to the centrifuge tube. After centrifugation, the blood separated into three layers (Figure 2A). The upper serum layer was collected using a syringe and placed on a sterile gauze, while the lower blood cell layer was discarded. The middle layer was identified as pure platelet-rich fibrin (P-PRF) gel and retained. This preparation protocol (3,000 rpm high-speed centrifugation without anticoagulants) conforms to the definition of P-PRF, which is featured with high platelet concentration and low leukocyte content. In this study, blood was collected intraoperatively, and P-PRF was prepared simultaneously with the surgery (Dohan Ehrenfest et al., 2009).

#### 2.4.3 Preparation of AP/CS/β-GP/PVA composite PRF scaffold

During the surgery, the AP/CS/β-GP/PVA scaffold was mixed with an equal volume of PRF using ophthalmic scissors and implanted into the bone defects of the experimental group (Figures 2B,C).

#### 2.4.4 Establishment of periodontal bone defect model

**FIGURE 3:**
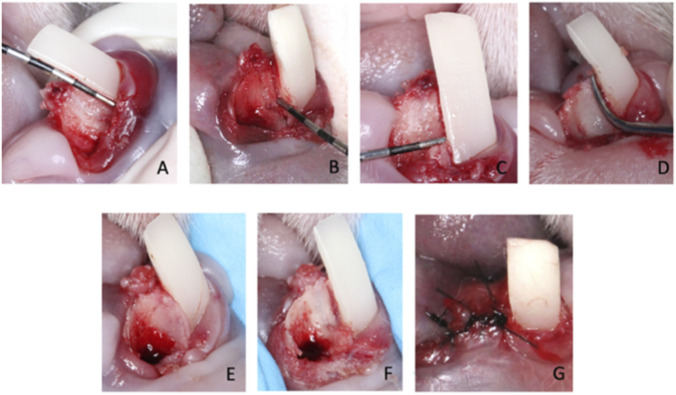
Establishment of periodontal bone defect model. **(A–C)** Standardized measurement of defect depth using a calibrated periodontal probe to ensure consistent preparation **(D)** Root surface debridement using a periodontal curette to remove residual periodontal ligament fibers and soft tissues from the root surface; **(E, F)** Creation of a 5-mm deep critical-sized alveolar bone defect; **(G)** Primary closure of the gingival flap with interrupted sutures.

All surgical procedures were performed by the same experienced operator to minimize individual variability. The standardized protocol was strictly followed: (i) the alveolar bone crest (ABC) was used as the unified and fixed anatomical reference for defect depth measurement; (ii) a low-speed round bur was utilized under continuous saline cooling; (iii) the buccal bone plate was removed horizontally by 2 mm to create a defect approximately 5 mm in depth below the ABC; and (iv) a 1-mm-thick bone layer was preserved on the mesiolingual aspect to prevent mandibular fracture. This model was selected based on the principle that bone defects exceeding this critical size (5 mm) do not undergo spontaneous healing and thus require intervention, enabling an effective evaluation of the osteogenic potential of the implanted materials (Takeuchi et al., 2023; Yu et al., 2025). Following root surface debridement and the creation of a histological reference notch, the respective materials were implanted into the defects for the AP/CS/β-GP/PVA composite PRF scaffold group, AP/CS/β-GP/PVA scaffold group, PRF group, AP/PVA scaffold group, and Bio-Oss® bone powder group, whereas no material was implanted in the blank control group. Finally, the gingival flap was sutured in all animals to ensure primary closure. Postoperatively, 4 × 10^5^ U of penicillin was intramuscularly injected once daily for 3 consecutive days to prevent infection (Figure 3).

#### 2.4.5 Sample collection and specimen processing

New Zealand White rabbits are classic and widely employed preclinical models for alveolar bone regeneration research. A 12-week observation duration has been extensively adopted in relevant studies of this species and is adequate to comprehensively evaluate bone regeneration, material degradation and bone remodeling (Sowmya et al., 2017; García-Lamas et al., 2023; Lee et al., 2025). At 4, 8, and 12 weeks post-surgery, three rabbits in each group were randomly selected and weighed. Sumianxin II injection was administered intramuscularly at the thigh at a dose of 0.2–0.3 mg/kg. Following the complete loss of pupillary reflex and attainment of adequate anesthetic depth, the rabbits were euthanized by air embolism via the ear marginal vein with air injection at a dose of 10∼20 mL/kg. The dental and periodontal tissue blocks in the surgical area were immediately harvested for subsequent imaging analysis. The specimens were then fixed in 4% formaldehyde solution and immersed in 10% ethylenediaminetetraacetic acid (EDTA) decalcification solution. After complete decalcification, the specimens were subjected to gradient ethanol dehydration and paraffin embedding. 5 μm-thick tissue sections were cut in the distal-mesial and buccal-lingual directions, with serial sections prepared from the buccal tissue.

### 2.5 Main observation indicators

#### 2.5.1 Macroscopic observation

Visual Inspection: Observe the wound healing and infection resistance at the surgical site in the rabbits, the color, shape, and quality of the gingiva (periodontal pocket), the height of the soft tissue for any absorption or recession, the formation of periodontal abscesses, and the absorption status of the materials.

#### 2.5.2 Radiological observation

After specimen collection at 4, 8, and 12 weeks post-surgery, X-ray examination was performed. Images were saved, and observations were made on the alveolar bone density and height, whether the bone hard line was visible, and the degradation status of the new gel scaffold, including changes in its volume and bone density.

#### 2.5.3 Histopathological observation

HE Staining:

- Deparaffinization and Rehydration: Mandibular bone tissue sections collected from rabbits at 4, 8, and 12 weeks post-surgery were deparaffinized by immersion in xylene I and xylene II for 10 min each. Subsequently, the sections were rehydrated through a graded alcohol series (100% I, 100% II, 95%, 85%, 70%) for 5 min each, followed by rinsing in distilled water (3 × 5 min).
- Sections were immersed in hematoxylin solution for 3–5 min to stain cell nuclei, followed by a brief rinse in tap water. Differentiation was performed using 1% acid alcohol (1% HCl in 70% ethanol) for a few seconds until the desired contrast was achieved, followed by washing in running water. Sections were then treated with a bluing solution (or saturated lithium carbonate/ammonia water) to restore the blue color of the nuclei and rinsed thoroughly.
- Eosin Staining: After ensuring the sections were free of excess water, they were immersed in eosin Y solution for 3–5 min to stain the cytoplasm and extracellular matrix.
- Dehydration, Clearing, and Mounting: Stained sections were dehydrated through a graded alcohol series (70%, 85%, 95%, 100% I, 100% II) for 2–5 min each to remove excess eosin and water. The sections were then cleared in xylene I and xylene II for 5 min each until transparent. Immediately after clearing, a drop of neutral resin was applied, and the sections were covered with a coverslip while still wet to prevent drying artifacts. Morphological features of the scaffold materials, newly formed bone, and periodontal ligament were observed and captured using a light microscope.

### 2.6 Quality control

In this study, experimental animals were assigned to groups using a randomized controlled design. Animal selection adhered to strict standardized criteria. Model establishment was supervised by experienced researchers, and all bone defects were created by a single operator to ensure consistency in defect size and morphology. To minimize measurement bias, all radiological and histomorphometric assessments were performed in a blinded manner, with examiners unaware of the group allocation during data analysis. Prior to data collection, all examiners underwent standardized training and calibration to harmonize evaluation criteria and operational procedures for image analysis and morphometric measurements. Intra-observer and inter-observer reliabilities were evaluated using the intraclass correlation coefficient (ICC); an ICC value >0.75 was considered indicative of excellent reliability, ensuring the stability and credibility of the measurements. Post-experiment, X-ray imaging and data acquisition were conducted by the same operator using consistent parameters. For histological analysis, three sections per animal were randomly selected at each time point. Images were captured and analyzed using ImageJ software, and results were expressed as mean values to minimize experimental variability.

### 2.7 Statistical analysis

Histological sections and radiological images were acquired and exported in TIFF format for analysis. Prior to measurement, spatial calibration was performed using Fiji software (Fiji Is Just ImageJ; NIH, Bethesda, MD, United States) to convert pixel dimensions into micrometers (µm) for histological images. For radiological images, pixel-to-distance calibration was applied, and display settings (brightness and contrast) were standardized across all samples to ensure consistent Region of Interest (ROI) definition and comparability of gray value measurements. All morphometric and densitometric measurements were performed in triplicate, and the mean values were used for subsequent analysis.

1. Alveolar Bone Area: The contour of newly formed alveolar bone within the defect area was manually outlined using the polygonal selection tool in Fiji.
2. Periodontal Ligament (PDL) Width: Measurements were taken at multiple standardized sites along the root surface. The measurement line was oriented perpendicular to both the root surface and the alveolar bone crest.
3. Degree of Bone Mineralization: Regions of interest (ROIs) of consistent size were defined in the central defect area, the marginal defect area, and the ipsilateral native bone tissue on radiological images. The mean gray value for each ROI was determined using Fiji. The representative gray value for the defect region was calculated as the average of the central and marginal areas. The relative bone mineralization degree was then expressed as the ratio of this defect gray value to that of the ipsilateral native bone.

Statistical analyses were performed using SPSS Statistics version 29.0. Continuous variables were expressed as mean ± standard deviation (SD). Data normality and homogeneity of variance were assessed using the Shapiro-Wilk test and Levene’s test, respectively. One-way analysis of variance (ANOVA) was employed to compare the new alveolar bone area, PDL width, and relative bone mineralization ratio among groups at each time point. When a significant main effect was detected (P < 0.05), *post hoc* pairwise comparisons were conducted using the Bonferroni correction. A P-value of less than 0.05 was considered statistically significant.

## 3 Results

### 3.1 Macroscopic observation

**FIGURE 4:**
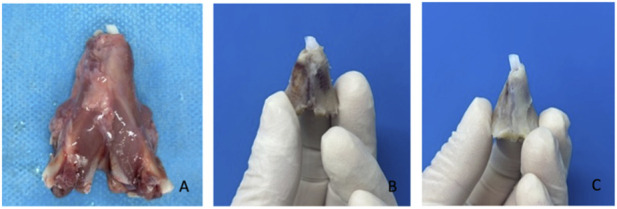
**(A,B)** AP/CS/β-GP/PVA composite PRF scaffold material group **(C)** blank group.

All animals showed good wound healing post-surgery, with no signs of infection or material exposure. In the experimental groups, the gingival color, shape, and texture were normal, and the gingival sulcus depth did not exceed 1 mm. In the blank control group, the alveolar bone height decreased at 8 weeks post-surgery, and gingival recession of 2–3 mm was observed (Figure 4).

### 3.2 Radiological observation

#### 3.2.1 At 4 weeks post-surgery

**FIGURE 5:**
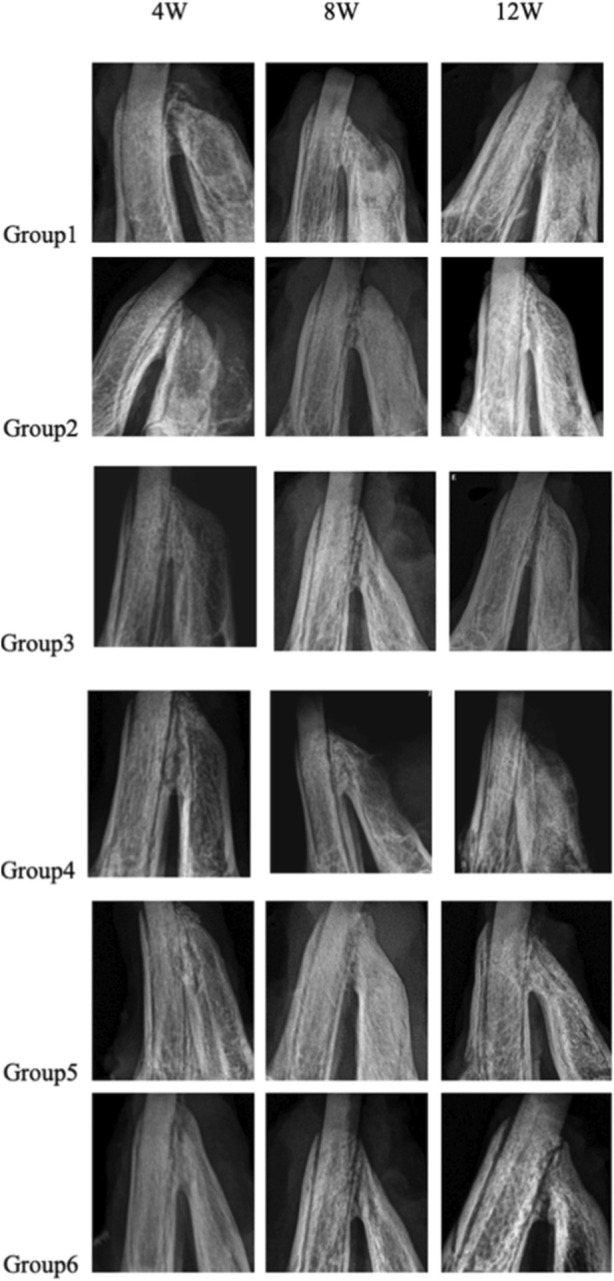
X-ray observation at different time points for each group. Group 1: AP/CS/β-GP/PVA Composite PRF Scaffold Material, Group 2: AP/CS/β-GP/PVA Scaffold Material, Group 3: PRF, Group 4: AP/PVA Scaffold, Group 5: Bio-Oss® bone powder, Group 6: Blank Group.

Group 1: New bone trabeculae were observed at the margin of the bone defect, and partial osseous continuity was established at the defect border; Group 2: Bone remodeling had initiated, characterized by heterogeneous alveolar bone density with mixed patchy high-density opacities and low-density areas, and the alveolar bone width appeared irregular; Group 3: The defect area presented as a homogeneous low-density region with mild resorption at the alveolar bone margin, and the alveolar ridge width was slightly narrower than that of the surrounding host bone; Group 4: A linear high-density opacity representing new bone was observed around the defect, while the alveolar bone adjacent to the defect remained predominantly low-density; Group 5: The bone defect was filled with granular radiopaque scaffold material, while the peripheral alveolar bone exhibited low-density changes; Group 6: The extraction socket appeared as a distinct radiolucent area, with no evidence of high-density new bone formation (Figure 5).

#### 3.2.2 At 8 weeks post-surgery

Group 1: The width of the cervical third of the alveolar bone was slightly narrowed. Overall bone density increased significantly compared to 4 weeks. Osseous union was achieved across the defect area, with no distinct radiolucent areas observed; Group 2: Bone tissue at the defect margin coalesced into continuous sheets. The width and height of the alveolar bone remained relatively intact, and bone density appeared more uniform than at 4 weeks; Group 3: The alveolar bone width was significantly narrower than that of the surrounding host bone. Although bone density in and around the defect increased compared to 4 weeks, partial continuous linear radiolucencies persisted within the defect. The overall density of the defect area remained lower than that of the adjacent native bone; Group 4: Marked alveolar bone resorption with irregular margins was observed. Continuous high-density opacities representing new bone were visible in the defect area, accompanied by localized remnants of granular scaffold material; Group 5: No obvious abnormalities were noted in alveolar bone width or height. However, low bone density with localized radiolucencies was observed near the cervical region of the defect. Overall bone density increased compared to 4 weeks; Group 6: Significant resorption of alveolar bone width and height was evident, particularly in width. Sparse new bone trabeculae formed within the defect, and the size of the radiolucent area decreased compared to 4 weeks (Figure 5).

#### 3.2.3 At 12 weeks post-surgery

Groups 1 and 2: Overall bone density was higher than in the other groups. The defect areas exhibited radiopaque structures resembling native bone tissue. Although slight heterogeneity persisted, the density approached that of the surrounding host bone. Continuous high-density opacities were observed across the defect without distinct radiolucencies. Notably, Group 1 demonstrated superior preservation of alveolar bone width compared to Group 2; Group 3: The alveolar bone displayed a density gradient, with higher density centrally and lower density peripherally. Alveolar bone width improved compared to 4 and 8 weeks. Continuous linear radiopacities were visible within the defect, while bone density gradually decreased from the defect center toward the surrounding host bone; Group 4: The marginal morphology of the alveolar bone was more regular than at earlier time points. Bone density was second only to Groups 1 and 2. Narrowing of the alveolar width was mild and limited to the cervical third; Group 5: Continuous radiopacities representing new bone were visible in the defect area, accompanied by localized granular remnants. Bone density gradually decreased from the defect margin toward the center; Group 6: Alveolar bone density remained heterogeneous with localized radiolucencies. Bone remodeling was ongoing, and the extent of the radiolucent area within the defect further decreased compared to 4 and 8 weeks (Figure 5).

### 3.3 Histopathological observation (hematoxylin-eosin staining)

#### 3.3.1 Morphological observation of newly formed bone tissue

##### 3.3.1.1 At 4 weeks post-surgery

**FIGURE 6:**
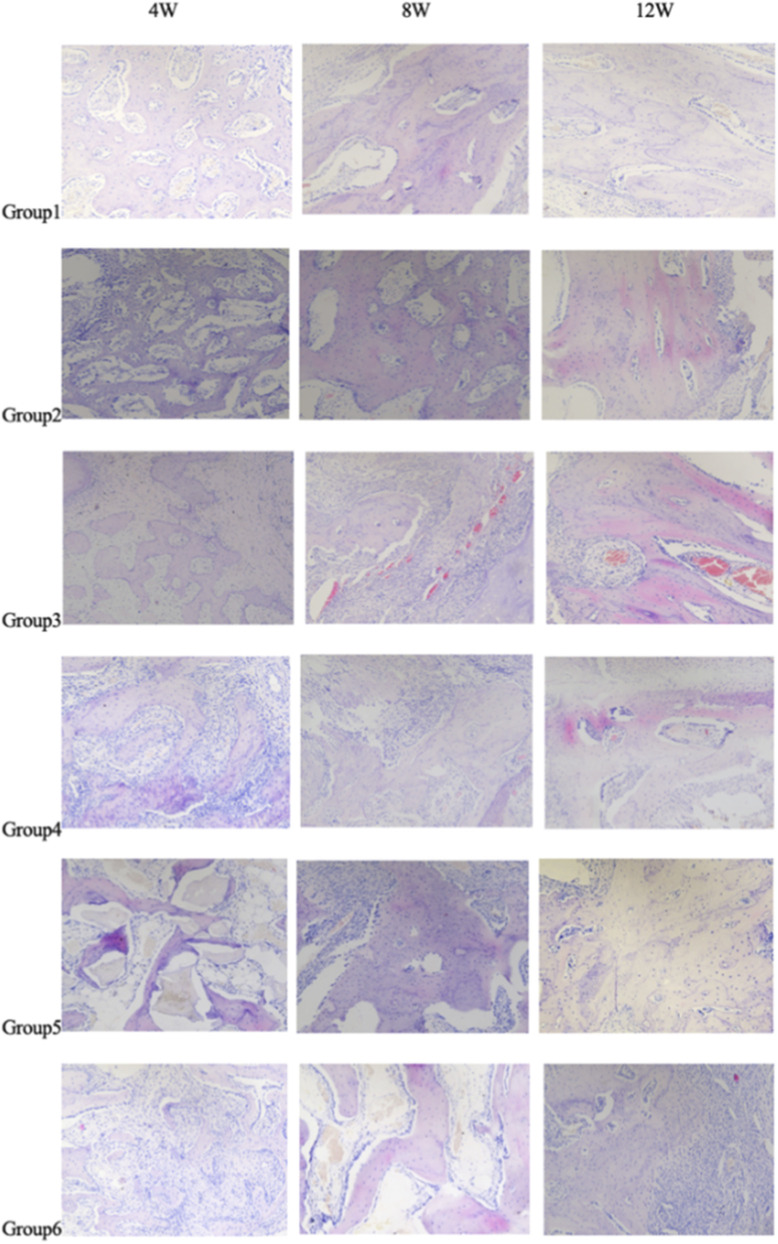
HE staining observation at different time points for each group (x100). Group 1: AP/CS/β-GP/PVA Composite PRF Scaffold Material , Group 2: AP/CS/β-GP/PVA Scaffold Material, Group 3: PRF, Group 4: AP/PVA Scaffold, Group 5: Bio-Oss® bone powder, Group 6: Blank Group.

Group 1 exhibited active bone remodeling. The newly formed bone was mainly composed of mature woven bone, and its trabeculae were interconnected into a three-dimensional reticular structure with high porosity. In these pores, osteoblasts were arranged concentrically around blood vessels to form obvious ossification centers, and deposited osteoid layer by layer. Osteoblasts surrounding osteoprogenitor cells showed high functional activity, which promoted the continuous deposition and fusion of new bone tissue. Around these regions, basophilic cells radiated from dense clusters to sparse areas, accompanied by extensive collagen fiber formation. Multiple ossification centers rich in blood vessels were observed, indicating significant osteogenic activity; Group 2 presented an obvious reticular osteogenic trend. Nevertheless, its trabecular density was lower than that of Group 1, and the number of new blood vessels was also reduced. New bone was deposited in fibrous tissue, showing partial sheet-like structures and delicate reticular patterns, with a somewhat irregular trabecular arrangement; A small amount of undegraded material was observed in Group 3. The surrounding trabeculae were in the early formation stage, presenting as fine reticular bone, and the area of new bone was quite limited; Group 4 showed massive proliferation and hyperactivity of osteoblasts, with trabecular widening and partial regional continuity. However, obvious inflammatory cell infiltration could be seen, and both the maturity of new bone tissue and the continuity of trabeculae were inferior to those of Groups 1 and 2; Group 5 displayed pale eosinophilic sheet-like structures, which acted as scaffolds for osteoblast adhesion. Osteoblasts were densely distributed on the surfaces and pores of bone powder particles, and directly secreted osteoid that mineralized into new trabeculae around the particles. Osteogenic activity was detected, and bone powder particles were gradually integrating with the newly formed bone tissue; Group 6 had sparse and scattered trabeculae. The tissue contained inflammatory cells and only a few osteoblasts, with no obvious formation of mature bone tissue (Figure 6).

##### 3.3.1.2 At 8 weeks post-surgery

Group 1 maintained high osteoblastic activity. The newly formed woven bone coalesced into continuous sheets with typical lacunar structures, and part of the sheet-like bone turned into mature lamellar bone. The number of ossification centers remained stable, osteoid was sufficiently mineralized, and pore sizes decreased uniformly. The number of bone lacunae and new blood vessels was obviously higher than that at week 4, with massive formation of osteoid and new bone. The scaffold material was still involved in bone remodeling, and its partially degraded products formed collagen fibers that integrated closely with new bone; Group 2 also showed active osteoblasts. The area of reticular bone was larger than that at week 4; the trabecular arrangement became denser and connected widely into large sheets. Neovascularization was increased compared with week 4, and the new bone area kept expanding to gradually fill the original implant site. Some sheet-like bone matured into lamellar bone, and osteoid in ossification centers was fully mineralized; Group 3 exhibited obvious sheet-like and reticular osteogenesis. Fibroblasts were regularly arranged in relatively thick layers. The central defect was rich in blood vessels, and the structure of new bone trabeculae was gradually improved; Group 4 presented massive new bone formation that integrated tightly with the surrounding alveolar bone, and osteoblastic activity persisted. New bone trabeculae were distributed in sheets, but some regions were discontinuous. The maturity of bone tissue was higher than that at week 4; Group 5 showed that bone powder particles were widely wrapped by abundant new bone tissue. Osteoblasts remained highly active, and bone volume was significantly increased compared with week 4. Blood supply was favorable, and new bone connected into continuous sheets and gradually matured; Group 6 had more new bone than that at week 4, with active osteoblasts and osteoclasts. However, fibrous connective tissue was still scattered in the lesion. The new bone was the least mature among all groups, with thin and sparsely distributed trabeculae (Figure 6).

##### 3.3.1.3 At 12 weeks post-surgery

Group 1 formed extensive mature sheet-like and lamellar bone. Mature bone lacunae spread to the periphery, forming dense lamellar bone with obvious reversal lines, and typical Haversian systems (osteons) were observed. A great number of mature new blood vessels surrounded the new bone tissue; Group 2 developed massive sheet-like bone with embedded new blood vessels, which guaranteed sufficient blood supply and relatively high tissue maturity. No typical Haversian systems could be found in this group; Group 3 was mainly characterized by sheet-like bone formation. The defect center contained abundant new blood vessels and a dense vascular network, but both the osteogenic area and bone tissue density were lower than those in Groups 1 and 2; Group 4 still showed active osteoblasts. The bone trabeculae became more mature with distinct ossification centers. The implanted material degraded completely, and plenty of collagen fibers appeared in the degraded areas, which integrated closely with the new bone; Group 5 presented extensive mature lamellar bone at 12 weeks. Typical Haversian systems emerged in the bone tissue, and bone remodeling tended to be steady, though the lamellar bone maturity was slightly lower than that in Group 1; Group 6 was in the process of continuous bone remodeling, and the new bone became more mature with partial connection. Nevertheless, the volume, maturity and area of new bone were the lowest among all groups (Figure 6).

#### 3.3.2 Morphological characterization of the scaffold materials

**FIGURE 7:**
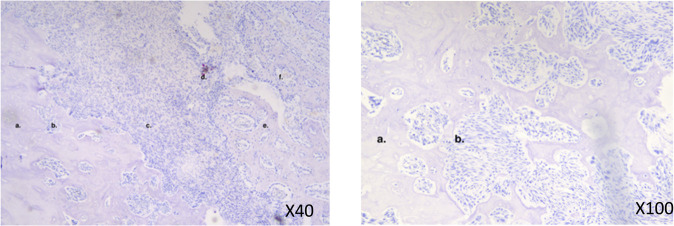
Microscopic observations of the AP/CS/β-GP/PVA composite PRF scaffold at different magnifications (Group 1, 4 weeks).

**FIGURE 8:**
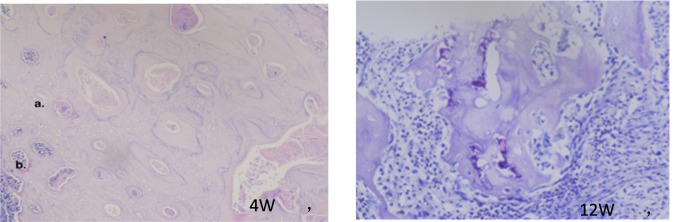
Observation of the AP/CS/β-GP/PVA scaffold at different time points and under different microscopic magnifications (Group 2).

**FIGURE 9:**
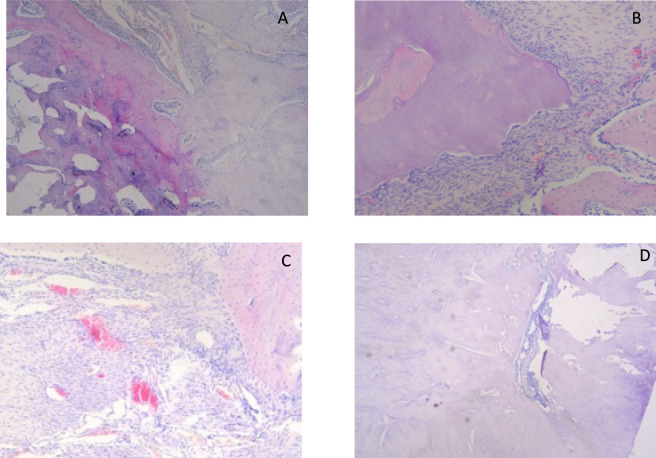
Morphological characterization of the transition toward bone maturation, cellular organization, scaffold network architecture, and material integration (Group 1, X100) **(A)** Partial transition from immature bone to mature bone, 8w **(B)** osteoblasts and fibroblasts aligned in a parallel gradient along the material–new bone interface, 8w; **(C)** a three-dimensional fibroblast scaffold network, 8w **(D)** integration of the material with dentin, 4w.

**FIGURE 10:**
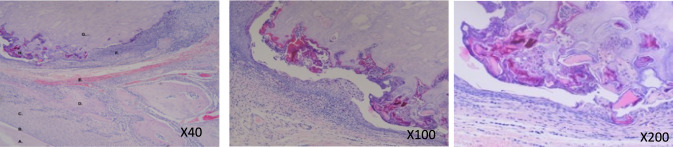
Microscopic observations of the AP/CS/β-GP/PVA-PRF composite scaffold at different magnifications (Group 1, 12 weeks). (A) Dentin; (B) Cementum; (C) Periodontal ligament; (D) Newly formed alveolar bone; (E) Neovasculature; (F) Cellular region; (G) Material region (residual, not fully degraded scaffold); (H) Scaffold degradation region.

**FIGURE 11:**
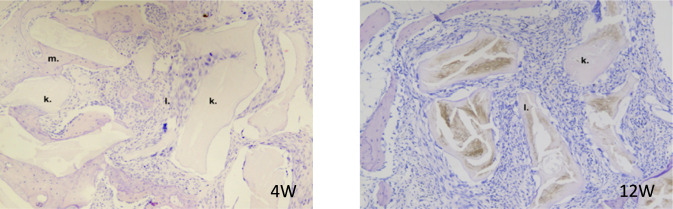
Observation of Bioss bone powder group at ×100 magnification (Group 5) (k) Bio-Oss bone powder area; (l) Cellular zone; (m) bone trabeculae.

HE staining revealed a loose three-dimensional structure of the composite hydrogel scaffolds in Groups 1 and 2, and the porous feature was especially prominent in Group 2 (Figures 7, 8). Cell infiltration was observed around the materials and within their pores. Dynamic observation at 4, 8, and 12 weeks postoperatively demonstrated that the scaffolds in Groups 1 and 2 degraded gradually over time. Functionally active osteoprogenitor cells, fibroblasts, and collagen fibers were found in both the degraded regions and the remaining porous areas. At the same time, newly formed fibrous connective tissue gradually occupied the defect as the scaffolds degraded. In early histological sections of Group 1, numerous osteoblasts and fibroblasts aligned in a parallel gradient along the material–new bone interface, with abundant capillaries distributed within this region (Figures 8, 9B). Sections at the same time point also showed a fibroblastic repair network near the new bone, which contained mature neovessels and osteoblasts (Figure 9C). At 12 weeks postoperatively, some undegraded scaffolds remained and were undergoing gradual degradation in the defect sites of Groups 1 and 2. Meanwhile, abundant blood vessels were observed around the scaffold materials in Group 1 (Figures 8, 10). In addition, histological sections of Group 1 showed obvious integration between dentin and the composite hydrogel (Figure 9D). In comparison, Group 3 also displayed some pore structures of different sizes, but massive degradation was observed as early as 4 weeks postoperatively. In Group 5, eosinophilic granular materials were distributed in sheet-like aggregates, adhering closely to the newly formed bone and surrounded by osteoblasts. Notably, a considerable amount of undegraded material wrapped by cells could still be seen at 12 weeks postoperatively (Figure 11).

**FIGURE 12:**
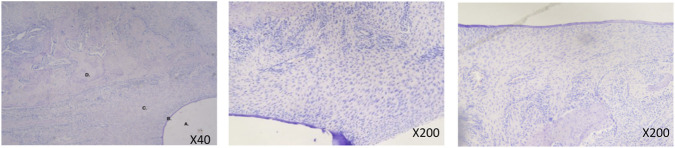
Periodontal ligament in Group 1 observed at different magnifications (8 weeks).

#### 3.3.3 Morphological observation of the periodontal ligament and cementum

**FIGURE 13:**
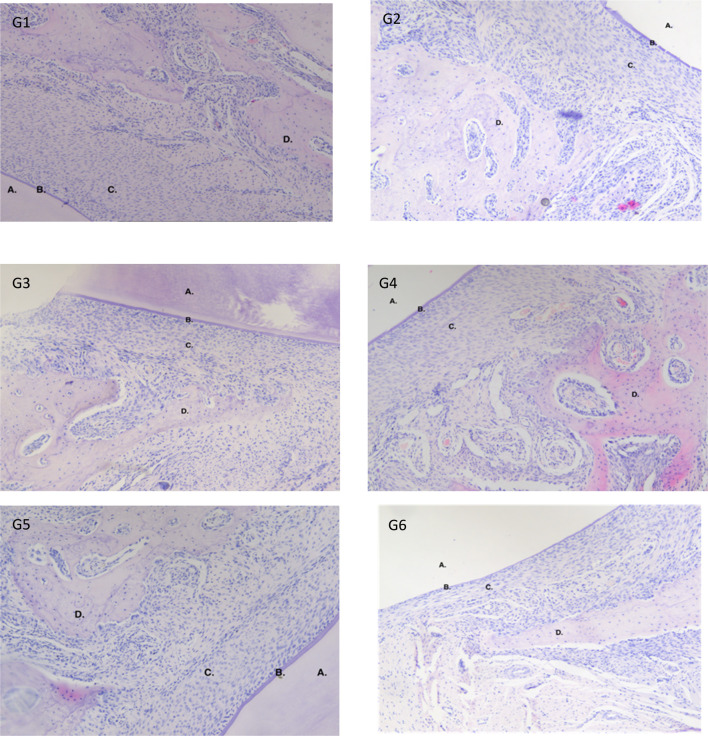
Microscopic observation of the periodontal ligament in Groups 1–6 at 12 weeks (×100 magnification). (A) Dentin region; (B) Cementum; (C) Periodontal ligament; (D) Alveolar bone.

At 12 weeks post-operation, histological sections of Group 1 showed significantly widened periodontal ligament (PDL) with uniform thickness. The fibers were arranged in a functionally regular pattern, similar to the attachment morphology of normal Sharpey’s fibers, and the cementum was visibly thickened. The PDL widening was particularly prominent in the apical region, with coordinated regenerative morphology among the PDL, cementum, and surrounding bone tissue. Overall, Group 1 achieved the best periodontal tissue regeneration, approaching a normal physiological state. Group 5 also exhibited significantly widened PDL with uniform thickness, and the cementum was visibly thickened. Although the functional arrangement of the fibers was less regular than that in Group 1, the overall condition remained relatively favorable. The periodontal tissue regeneration in Group 2 was second only to Groups 1 and 5. Histological observation revealed well-aligned PDL fibers, and the cementum repair was good. In Group 3, proliferation of fibrous tissue and vascular-like structures was observed in the PDL, but the fibers were poorly organized, randomly distributed, and lacked obvious functional arrangement characteristics. Group 4 had a similar degree of PDL fiber organization to Group 2, with its fibers uniformly distributed, but the cementum thickening was less pronounced than in Group 2. Group 6 had the poorest periodontal tissue repair outcome: the PDL fiber layer was thin and loose with no significant widening, the cementum repair was poor and failed to form a uniformly thickened structure, and the coordination of periodontal tissue regeneration was inadequate (Figures 12, 13).

### 3.4 Comparison of new alveolar bone area, periodontal ligament width and bone mineralization degree among groups at different time points

Statistical analysis at 4, 8, and 12 weeks postoperatively showed that all three indicators, including newly formed alveolar bone area, periodontal ligament (PDL) width and bone mineralization degree, increased in a time-dependent manner in all groups. One-way ANOVA also verified highly significant differences among groups at each time point (P < 0.001). Detailed comparisons of each indicator are provided as follows.

#### 3.4.1 New alveolar bone area

Group 1 showed the largest newly formed alveolar bone area at all time points, with significant differences compared with all other groups (*P < 0.05). Group 2 had the second-largest newly formed alveolar bone area and was significantly higher than Groups 3, 4, 5 and the blank control group (Group 6) (#P < 0.05). Group 5 presented better outcomes than Groups 3 and 4. Although its mean alveolar bone area value was similar to that of Group 2 at 8 weeks (P > 0.05), it was notably lower than Group 2 at both 4 and 12 weeks (#P < 0.05). Groups 3 and 4 had no significant difference in newly formed alveolar bone area at any time point. Both groups were significantly better than Group 6 but were significantly weaker than Groups 1, 2 and 5. The blank control group (Group 6) showed the smallest newly formed alveolar bone area throughout the observation period (Table 1).

**TABLE 1:** Alveolar bone area (mm^2^) at different time points for each group.

Group	4 W	8 W	12 W
Group 1	1.76 ± 0.10	2.17 ± 0.19	2.81 ± 0.23
Group 2	1.49 ± 0.12*	1.70 ± 0.14*	2.36 ± 0.03*
Group 3	1.21 ± 0.14*#	1.41 ± 0.16*#	1.75 ± 0.15*#
Group 4	1.19 ± 0.14*#	1.37 ± 0.20*#	1.81 ± 0.20*#
Group 5	1.23 ± 0.08*#	1.75 ± 0.08*▲●	2.03 ± 0.01*#▲
Group 6	0.85 ± 0.10*#▲●▼	1.04 ± 0.09*#▲●▼	1.30 ± 0.17*#▲●▼
F	14.121	22.546	40.778
P	<0.001	<0.001	<0.001

#### 3.4.2 Periodontal ligament (PDL) width

Group 1 formed the widest PDL at all time points, which was significantly larger than that in other groups (*P < 0.05). Different from the bone area results, Group 5 formed the second-widest PDL and was significantly wider than Groups 2, 3, 4 and 6 at all time points (P < 0.05). A dynamic change in PDL width was observed between Group 4 and Group 2: their PDL widths were similar at 4 weeks (P > 0.05), but Group 4’s PDL width grew much more slowly afterward, leading to a significantly narrower PDL width than that of Group 2 at 8 and 12 weeks (#P < 0.05). Group 3 had a significantly wider PDL than Group 6 but narrower than Groups 1, 2, 4 and 5. Group six remained the narrowest in PDL width across all time points (Table 2).

**TABLE 2:** Periodontal ligament width (μm) at different time points for each group.

Group	4 W	8 W	12 W
Group 1	109.88 ± 8.32	126.44 ± 8.48	143.33 ± 9.20
Group 2	88.82 ± 3.83*	108.44 ± 4.71*	122.15 ± 5.74*
Group 3	66.13 ± 7.34*#	79.99 ± 6.18*#	85.38 ± 4.44*#
Group 4	83.58 ± 3.18*▲	90.04 ± 3.76*#▲	93.85 ± 5.73*#
Group 5	97.06 ± 3.25*^▲●^	119.77 ± 2.76^#▲●^	130.31 ± 1.78*^▲●^
Group 6	45.49 ± 5.73*#▲●▼	57.98 ± 8.76*#▲●▼	67.39 ± 3.92*#▲●▼
F	46.649	59.719	76.083
P	<0.001	<0.001	<0.001

#### 3.4.3 Osteogenic mineralization degree

Group 1 maintained the highest mineralization level at all time points, significantly higher than that in other groups (*P < 0.05). Group 2 ranked the second, and showed a similar mineralization level to Group 5 with no statistical difference (P > 0.05). Both groups were significantly superior to Groups 3, 4 and 6 (#P < 0.05). Further comparison indicated that Group 5 was significantly higher than Group 3 at all time points (▲P < 0.05), and only significantly exceeded Group 4 at 12 weeks (●P < 0.05), with similar values at 4 and 8 weeks (P > 0.05). In addition, Group 4 had significantly higher mineralization than Group 3 (▲P < 0.05). Both Groups 3 and 4 were better than Group 6 but lower than Groups 1, 2 and 5. Group six remained the lowest in bone mineralization throughout the observation period (Table 3).

**TABLE 3:** Osteogenic mineralization degree at different time points for each group.

Group	4 W	8 W	12 W
Group 1	0.596 ± 0.017	0.713 ± 0.008	0.764 ± 0.011
Group 2	0.523 ± 0.032*	0.611 ± 0.017*	0.699 ± 0.010*
Group 3	0.456 ± 0.020*^#^	0.556 ± 0.003*^#^	0.592 ± 0.008*^#^
Group 4	0.489 ± 0.003*^#▲^	0.595 ± 0.017*^▲^	0.668 ± 0.011*^#▲^
Group 5	0.501 ± 0.010*^▲^	0.616 ± 0.013*^▲^	0.691 ± 0.014*^▲●^
Group 6	0.357 ± 0.013*^#▲●▼^	0.410 ± 0.020*^#▲●▼^	0.446 ± 0.017*^#▲●▼^
F	57.159	143.192	247.250
P	<0.001	<0.001	<0.001

*P < 0.05 compared with Group 1; #P < 0.05 compared with Group 2; ▲P < 0.05 compared with Group 3; ●P < 0.05 compared with Group 4; ▼P < 0.05 compared with Group 5.

## 4 Discussion

Scaffold materials play a vital role in tissue engineering, as the microenvironment they provide can significantly regulate cell adhesion, proliferation, and differentiation (Carvalho et al., 2008). With continuous advances in tissue engineering, scaffold microstructure has become an increasingly important research focus. An ideal scaffold should possess a suitable porous architecture and efficient mass transport properties. Its well-developed interconnected pore network guarantees smooth exchange of gases, nutrients, and metabolic wastes within the scaffold, thus providing a stable growth microenvironment for seeded cells, enhancing cell viability and biological activity, and laying a solid foundation for stem cell differentiation and tissue regeneration (Song et al., 2023; Mukasheva et al., 2024).

Based on the above structural and functional criteria for an ideal scaffold, histological sections were used in this study to observe the microstructure of the composite scaffold and tissue regeneration. A loose three-dimensional structure was observed in the composite hydrogel scaffolds of Groups 1 and 2, which degraded gradually over time. Highly active osteoprogenitor cells, osteoblasts, fibroblasts, collagen fibers, and other osteogenesis-related components were found in both the scaffold degradation zones and the porous 3D framework, suggesting that scaffold degradation and new bone formation occurred simultaneously.

During the degradation of bone regenerative scaffolds, active osteoprogenitor cells and fibroblasts are preferentially recruited to the degradation zones and residual structures. These cells rapidly produce collagen fibers to form an early matrix network for bone regeneration, laying a foundation for subsequent mineralization (Kumar et al., 2019; Liu et al., 2021). Such fibrous tissue is secreted by fibroblasts infiltrating into the scaffold pores and, together with blue-stained spindle-shaped mesenchymal-like cells, establishes the early bone regeneration microenvironment. In the early post-implantation stage, the ingrowth of fibrous tissue into and around the scaffold pores provides a favorable microenvironment for the adhesion, migration, and engraftment of mesenchymal stem cells. In addition, osteoinductive scaffolds can effectively recruit host mesenchymal stem cells to migrate into the bone defect area (Rodriguez et al., 2014; Yoshida et al., 2015). As critical seed cells for bone regeneration, mesenchymal cells can directionally differentiate into osteoblasts under an osteoinductive microenvironment, participate in bone matrix formation and mineralization, and ultimately promote new bone regeneration (Zeng et al., 2024). The composite scaffold fabricated in this study may also provide a beneficial microenvironment for bone regeneration through similar mechanisms.

Collectively, these findings demonstrate that the scaffold prepared in this study exhibits the essential characteristics of an ideal tissue-engineered scaffold, including an interconnected 3D porous structure, favorable degradability, and distinct osteoinductive potential, thereby providing a favorable microenvironment for bone regeneration.

In comparison, Group 5 showed granular and sheet-like eosinophilic materials, which were morphologically distinct from the interconnected 3D porous scaffold in Group 1. Owing to its natural osteoconductivity, Bio-Oss bonded directly with the host bone. Osteoblasts attached and wrapped around the material, yet cell infiltration was mostly limited to the material surface and gaps between particles, with only a few cells invading its inner micropores. At 12 weeks after surgery, most of the material still had not degraded, indicating a certain level of inertness. The osteoinductive potential of Bio-Oss bone powder is still debated in the literature. Some studies suggest that residual collagen and other components may help promote new bone formation. Other researchers, however, argue that thorough deproteinization removes key bioactive factors required for osteoinduction. Bio-Oss therefore only supports bone repair indirectly through osteoconduction, with limited ability to actively drive periodontal tissue regeneration (Ferraz, 2023).

In contrast, as observed by HE staining, the porous structure of the scaffold in Group 4 was less well-organized compared with Groups 1 and 2, and cellular infiltration was also relatively limited. Combined with intraoral radiographic imaging and statistical analysis, significant differences in bone mineralization were identified among groups. We therefore speculate that the absence of CS/β-GP modification may account for its suboptimal osteogenic performance. Group 6 relied solely on the body’s natural healing capacity. At all postoperative time points, the bone defect was mainly filled with fibrous connective tissue, with scarce new bone formation and insufficient periodontal tissue reconstruction, failing to achieve effective repair. These results further indicate that the composite scaffold used in this study plays a positive role in bone and periodontal tissue regeneration.

**FIGURE 14:**
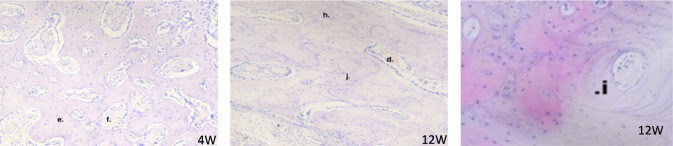
High-magnification observations of the AP/CS/β-GP/PVA-PRF composite scaffold at different time points (Group 1) (a) Material region (a loose three-dimensional structure is visible); (b) scaffold degradation region; (c) cellular transition zone (containing osteoprogenitor cells, osteoblasts, fibroblasts, etc., arranged in alternating layers parallel to the interface between the scaffold and the newly formed bone surface); (d) newly formed blood vessels; (e) woven bone; (f) osteogenic center (containing a single, orderly layer of osteoblasts and neovasculature); (g) lamellar bone; (h) bone lacunae (showing osteoid mineralization); (i) Haversian system; (j) lamellar bone.

Group 1 exhibited an orderly progression of bone formation from the early stage to late maturation. By 4 weeks, it already exhibited woven bone, with layered deposition of osteoid that partially coalesced into continuous areas. By 8 weeks, osteoid mineralization was evident; pores became more uniformly reduced in size, lacunae increased in number, and extensive plate-like bone formed, reflecting increased bone maturity and a transition of some immature bone toward mature bone. At 12 weeks, mature lacunae extended outward to form dense lamellar bone, with clear reversal lines and a visible Haversian system, a hallmark of robust bone regeneration (Figure 14) (Chang and Liu, 2022). During osteogenesis, one of the most characteristic positive indicators is the appearance of osteoblasts and fibroblasts arranged in a gradient on the material surface, accompanied by abundant capillaries; this pattern is considered most desirable when it emerges in the early to mid stages (Ros-Tárraga et al., 2016). Consistent with this, a large number of osteoblasts and fibroblasts were observed in Group 1 at 4 and 8 weeks, arranged in a graded manner parallel to the interface between the scaffold and the newly formed bone surface, with rich capillary networks embedded within the structure. In sections from the same time points, a three-dimensional fibroblast network was also observed near newly formed bone, containing mature neovasculature and osteoblasts. This structure, another positive sign of bone regeneration, may serve as a temporary osteogenic framework (Wang H. et al., 2024; Xu et al., 2025). These observed phenomena provide key support for the orderly progression and high maturity of osteogenesis in Group 1.

In contrast, although trabecular bone structure was observed in Group 5 at 4 weeks postoperatively, its osteogenic timeline and tissue morphology differed markedly from those of Group 1, with disorganized and irregular trabecular arrangement. By 12 weeks postoperatively, even though lamellar bone tissue had formed in Group 5, its bone maturity was still slightly inferior to that of Group 1, and its bone mineralization degree was significantly lower than that of Group 1 at all time points (P < 0.05).

Group 3 exhibited inferior osteogenic morphology, osteogenic progression and new bone area compared with Groups 1 and 2, but showed distinct advantages in angiogenesis, fibroblast proliferation and fibrous tissue formation at all time points. The application of PRF in vascular regeneration has been validated by numerous studies (Gollapudi et al., 2022). In the process of bone formation, new blood vessels can supply oxygen and nutrients to the local region, recruit osteoprogenitor cells and stem cells, and deliver signaling molecules and growth factors, providing critical support for bone repair (Lv et al., 2024).

In comparison between Groups 1 and 2, Group one was significantly superior to Group 2 in osteogenic morphology, osteogenic progression, new bone area, angiogenesis and fibrous tissue formation. Early establishment of a functional vascular network is therefore critical for bone regeneration in defect sites and should be considered in tissue-engineered bone design (Deng et al., 2025). As mentioned previously, PRF can effectively promote angiogenesis in the defect area. Sufficient early blood supply provides abundant oxygen and nutrients for osteoblasts and mesenchymal stem cells, facilitates cell proliferation, matrix synthesis and bone tissue mineralization, and further accelerates the process of bone regeneration. This is also consistent with the overall superior tissue regeneration performance observed in Group 1.

During periodontal tissue regeneration, premature scaffold degradation tends to cause tissue collapse and is not conducive to maintaining the space required for periodontal regeneration (Liang et al., 2020). At 12 weeks after surgery, residual undegraded scaffold could still be observed in the defect areas of Groups 1 and 2. In contrast, the scaffold in Group 3 degraded much faster, the lack of sustained mechanical support may compromise its late-stage osteogenic outcome to some degree.

Group 1 achieved satisfactory periodontal tissue reconstruction at 12 weeks postoperatively. The regenerated periodontal ligament presented obvious widening and uniform thickness, and the fibrous tissues were arranged in a functional pattern that resembled normal Sharpey’s fiber characteristics. Meanwhile, evident cementum thickening was observed in the repair area. Some sections from Group 1 showed favorable integration between dentin and the composite hydrogel, which may indicate a potential capacity to promote dentin repair and confirm good biocompatibility. At 12 weeks postoperatively, the periodontal ligament width in Group 1 was significantly superior to that in the other groups (P < 0.05). Although the Bio-Oss group also showed increased periodontal ligament width and relatively organized fibers, its overall periodontal structure and ligament thickness were markedly inferior to Group 1 (P < 0.05).

These results further suggest that the AP/CS/β-GP/PVA composite scaffold combined with PRF not only promotes high-quality bone regeneration, but also effectively guides the orderly repair of the periodontal ligament and cementum. With its potential to promote dentin formation and good biocompatibility, this scaffold demonstrates superior overall capacity for periodontal tissue regeneration.

Current research on biological scaffold materials increasingly emphasizes combining the advantages of natural and synthetic components. Using multi-material composite strategies, investigators seek to engineer scaffolds that couple high biocompatibility with adequate mechanical strength, thereby addressing the limitations of single-source materials and improving overall scaffold performance (Thirumalaivasan, 2025). Against this background, we investigated a composite approach integrating deer antler powder, a hydrogel matrix, and platelet-rich fibrin (PRF), hypothesizing that this formulation would be effective for periodontal tissue regeneration.

From the perspective of material synergy, as a cationic alkaline polysaccharide, CS possesses excellent biocompatibility. The amino groups on its molecular chain can bind to cell surface receptors, providing a favorable microenvironment for cell adhesion, proliferation and bone regeneration (Aguilar et al., 2019). As a thermosensitive gelation regulator, β-GP enables the system to form a three-dimensional porous network structure, and achieves stable release of active ingredients through its sustained-release property to avoid the burst release effect (Bhattarai et al., 2010; Rahmanian-Devin, Baradaran Rahimi and Askari, 2021). PVA effectively compensates for the shortcomings of pure CS/β-GP hydrogels, such as insufficient mechanical properties and tendency to swell and collapse (Liang et al., 2024). Its favorable film-forming ability and pore-regulating capacity further optimize the interconnected pores of the scaffold, creating favorable conditions for cell migration and nutrient exchange.

Deer antler contains calcium-phosphate inorganic phases, type I collagen, and antler polypeptides, which endow it with excellent osteoconductivity and osteogenic capacity to promote host cell osteogenic differentiation and bone matrix mineralization. (Li et al., 2024). The AP/CS/β-GP/PVA group achieved better bone regeneration than the AP/PVA group, suggesting that the introduction of CS/β-GP enhances the overall osteogenic efficiency of the scaffold by optimizing the microstructure and biological properties of the hydrogel carrier. This result is consistent with the well-documented functions of CS/β-GP in thermosensitive gelation, sustained release and structural stabilization.

As an autologous bioactive material, PRF exerts pro-angiogenic and periodontal tissue repair effects by releasing a variety of growth factors (De Lauretis et al., 2024). Obvious alveolar bone resorption and a slow increase in bone mineral density in the PRF-only group not only further confirm the rationality of combining the hydrogel carrier with PRF, but also prove that a stable scaffold structure is an important prerequisite for PRF to fully exert its biological activity.

Compared with the Bio-Oss positive control group, the composite scaffold in this study showed better performance in the timing of osteogenic initiation, bone tissue maturity and mineralization level. Although Bio-Oss has favorable osteoconductivity, it exhibits relatively weak bioactivity, lacks active osteoinductive capacity, and leaves a certain amount of material residue long after surgery, which may compromise the integrity of bone repair to some extent. The limited spontaneous repair ability of the blank control group further confirms the positive application value of the composite scaffold in periodontal bone defect repair.

In summary, the structural support provided by the AP/CS/β-GP/PVA hydrogel, the osteoconductive and osteogenic regulatory effects of AP, and the pro-angiogenic and periodontal tissue repair effects of PRF may act synergistically to improve the overall regenerative performance of the composite scaffold. This scaffold can initiate bone regeneration at an early stage, promote the maturation and remodeling of bone tissue at a later stage, help maintain alveolar bone morphology, and facilitate the orderly repair of periodontal ligament and cementum, providing a promising tissue engineering scaffold strategy for periodontal bone defect repair. However, the optimal composite ratio of AP/CS/β-GP/PVA and PRF remains to be further investigated. In addition, there are still several limitations in this study. The molecular mechanisms driving periodontal repair and angiogenesis were not further explored. The postoperative observation period was limited to 12 weeks, and the long-term stability of regenerated periodontal tissues and the continuous degradation of composite scaffolds remain to be clarified with longer follow-up. Meanwhile, the animal defect model could not fully mimic the complex inflammatory microenvironment of clinical periodontal lesions. Subsequent research will focus on these aspects to further improve the research rigor and depth.

## 5 Conclusion

In summary, combined with our previous *in vitro* findings, the AP/CS/β-GP/PVA-PRF composite scaffold presents good biocompatibility and favorable osteogenic capacity, significantly promoting new bone formation and periodontal healing compared with the control group. This hydrogel composite scaffold is a promising candidate for periodontal regeneration, conducive to alveolar bone reconstruction and periodontal ligament repair. However, the specific regulatory mechanisms of its regenerative effects remain unclear. Further studies are needed to explore the related signaling pathways and lay a foundation for its clinical application in the future.

## Data Availability

The original contributions presented in the study are included in the article/supplementary material, further inquiries can be directed to the corresponding authors.
